# Supplementary material for: Cell size-dependent mRNA transcription drives proteome remodeling
Source: Cell Rep. Author manuscript; Available in PMC 2026 Jul 13. (PMC13363025; doi:10.1016/j.celrep.2026.117488)
Supplement: 1 [file NIHMS2190739-supplement-1.pdf]

Cell Reports, Volume *45*

Supplemental information

Cell size-dependent mRNA

transcription drives proteome remodeling

Dong Shin You, Christopher H. Bohrer, Purva H. Rumde, Ioannis Sanidas, Matthew P. Swaffer, Daniel R. Larson, Josh E. Elias, Michael C. Lanz, and Jan M. Skotheim

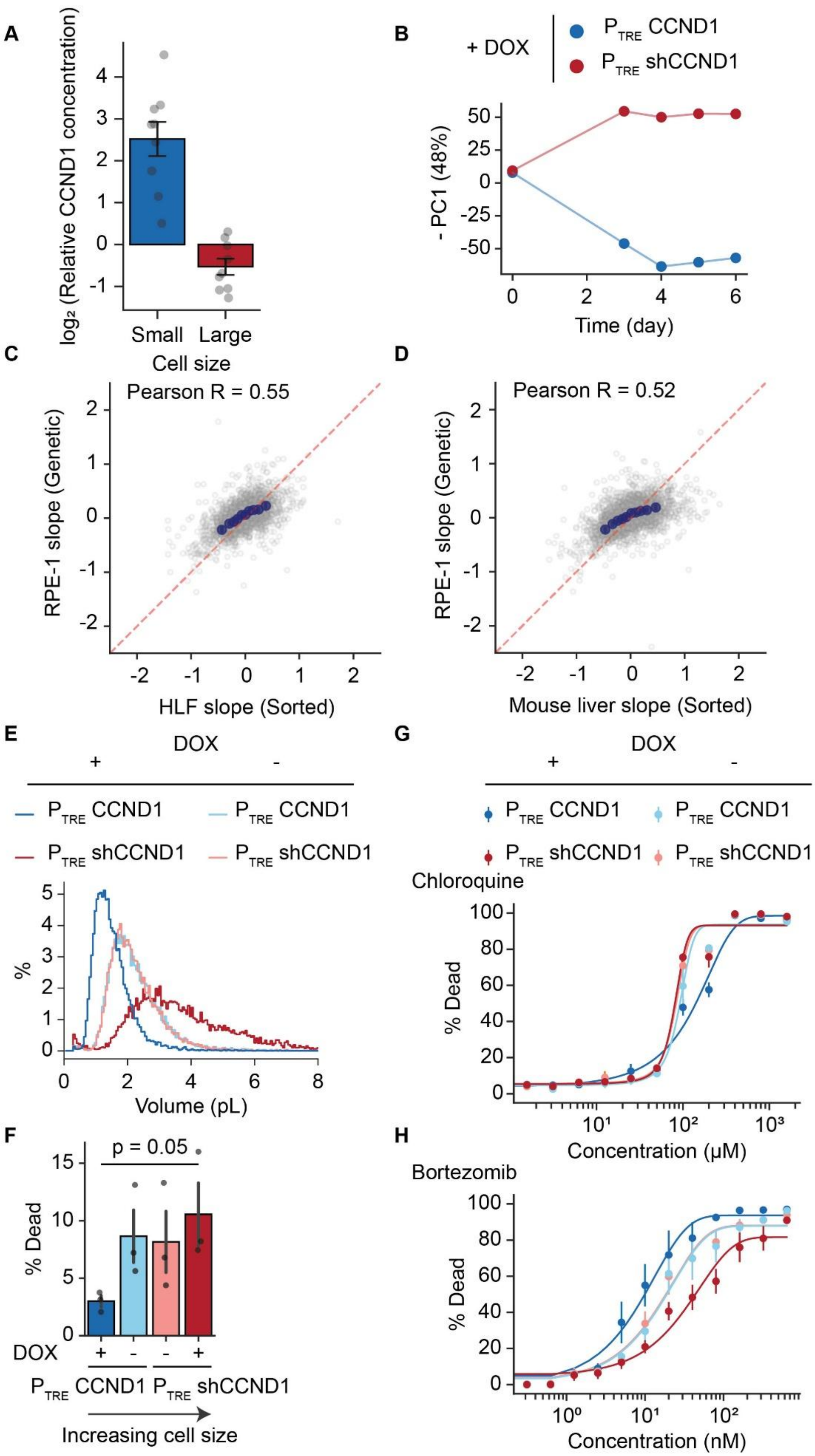
A
log₂ (Relative CCND1 concentration)
Small
Large
Cell size
B
+ DOX
P TRE CCND1
P TRE shCCND1
- PC1 (48%)
Time (day)
C
Pearson R = 0.55
RPE-1 slope (Genetic)
HLF slope (Sorted)
D
Pearson R = 0.52
RPE-1 slope (Genetic)
Mouse liver slope (Sorted)
E
DOX
+
-
P TRE CCND1
P TRE CCND1
P TRE shCCND1
P TRE shCCND1
%
Volume (pL)
F
p = 0.05
% Dead
DOX
P TRE CCND1
P TRE shCCND1
Increasing cell size
G
DOX
+
-
P TRE CCND1
P TRE CCND1
P TRE shCCND1
P TRE shCCND1
Chloroquine
% Dead
Concentration (µM)
H
Bortezomib
% Dead
Concentration (nM)

Figure S1. CCND1 manipulated cells display size-dependence in proteome composition and lysosome vulnerability, related to Figure 1.

A) Relative CCND1 peptide concentration as measured by mass-spectrometry following CCND1 knockdown or overexpression with doxycycline induction. Data was normalized to DMSO treated cells before log2 transformation such that uninduced cells have an expression level = 0. Averages of 8 high-quality peptides were taken. Error bars mark the standard error of the mean. **B)** Principal component analysis of P_{TRE} shCCND1 and P_{TRE} CCND1 cells' proteomes as measured by TMT-mass spectrometry before and after doxycycline induction. Cells were collected on days 0, 3,4,5, and 6. Comparison of the first principal component against time shows the cells reaching proteomic steady state after the 4th day of induction. **C)** Correlation of protein slopes derived using the CCND1 manipulation system in RPE-1 cells with protein slopes previously measured using size-sorted primary human lung fibroblasts (HLFs) (N = 2560 proteins)[S1,S2]. **D)** Same as **(C)** but for mouse primary liver cells from a previously published study (N = 3127 proteins)[S2]. Blue dots are averages of equal sized bins. **E)** Representative cell size distribution as measured by the Coulter counter for P_{TRE} shCCND1 and P_{TRE} CCND1 cells treated with doxycycline or DMSO for 5 days. **F)** Proportion of dying cells of the indicated conditions after 48 hour treatment of 50uM chloroquine, a lysosomotropic drug that perturbs lysosomal pH. Cell death was measured by Annexin V and a cell permeability dye. Baseline death rates from control (DMSO) conditions were subtracted to calculate death from chloroquine. Error bars represent the standard error of means. P-value shows outcome of unpaired t-test. N = 3 replicates. **G)** Proportion of dead cells across a wide dosage range after 3 days of chloroquine. P_{TRE} shCCND1 and P_{TRE} CCND1 cells induced with doxycycline or DMSO to new sizes were treated with the drug and assayed for cell death using SYTOX DeepRed and microscopy. Quantification of cell death was carried out using a custom nuclear segmentation algorithm (see methods). N = 3 replicates. **H)** Similar to **(G)**, but for 2 days of treatment with bortezomib. N=3 replicates. For both **G)** and **H)**, error bars represent the standard error of means.

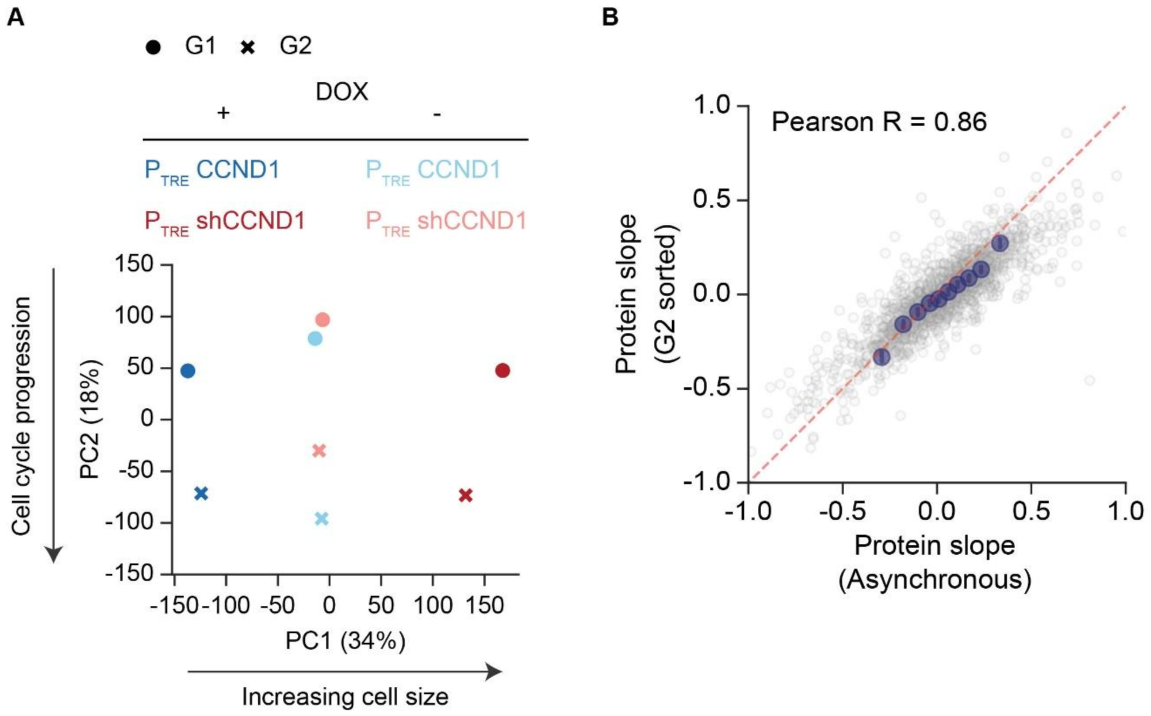
A
G1
G2
DOX
+
-
PTRE CCND1
PTRE CCND1
PTRE shCCND1
PTRE shCCND1
Cell cycle progression
PC2 (18%)
PC1 (34%)
Increasing cell size
B
Pearson R = 0.86
Protein slope
(G2 sorted)
Protein slope
(Asynchronous)

Figure S2. Cell size, not cell cycle, explains most of size-dependent proteome remodeling, related to Figure 2.

A) Principal component analysis of G1 and G2 sorted P_{TRE} shCCND1 and P_{TRE} CCND1 cells after 5 days of doxycycline or DMSO induction. Cells were sorted by cell cycle phase after DNA staining. Individual points show the proteome of indicated cells. Samples are separated by cell cycle and cell size, and samples in both cell cycle phases (G1 and G2) show the same pattern of change along PC1 (cell size).

B) Protein slopes as obtained from asynchronous conditions (as in **Figure 1F**) correlated against protein slopes from cells sorted in G2. The two samples are highly correlated with one another, indicating that cell cycle effects do not play a major role in size-dependent proteome remodeling. The limited number of proteins (N = 1882 proteins) reflects the shallow depth of the proteome analysis for this control experiment. Blue dots are averages of equal sized bins.

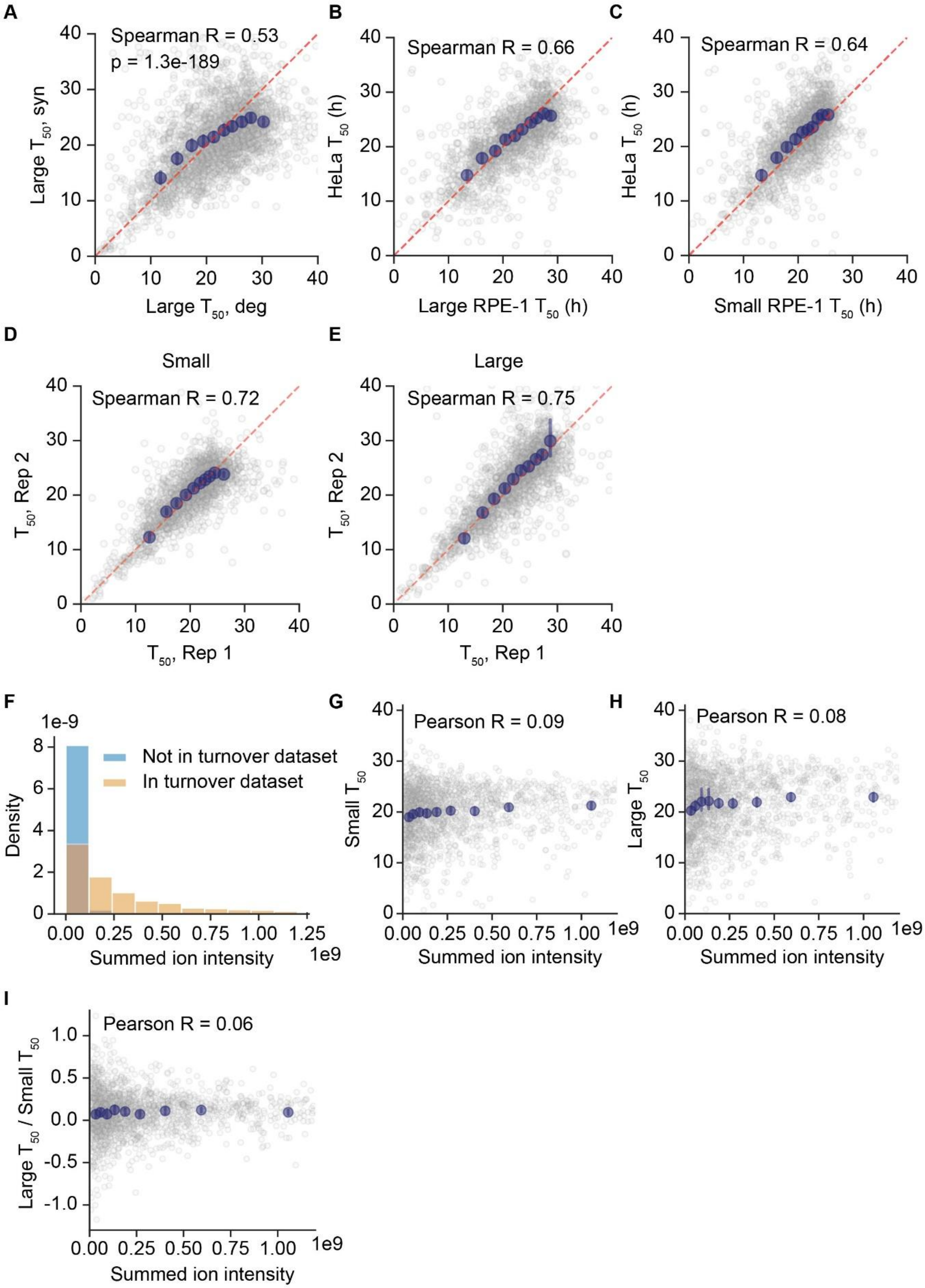

A
Spearman R = 0.53
p = 1.3e-189
Large T50, syn
Large T50, deg
B
Spearman R = 0.66
HeLa T50 (h)
Large RPE-1 T50 (h)
C
Spearman R = 0.64
HeLa T50 (h)
Small RPE-1 T50 (h)
D
Small
Spearman R = 0.72
T50, Rep 2
T50, Rep 1
E
Large
Spearman R = 0.75
T50, Rep 2
T50, Rep 1
F
1e-9
Density
Not in turnover dataset
In turnover dataset
Summed ion intensity
1e9
G
Pearson R = 0.09
Small T50
Summed ion intensity
1e9
H
Pearson R = 0.08
Large T50
Summed ion intensity
1e9
I
Pearson R = 0.06
Large T50 / Small T50
Summed ion intensity
1e9

Figure S3. Protein turnover is mostly stable across cell size, related to Figure 3.
A) Protein turnover comparison as calculated from incorporation (Large $T_{50,\ syn}$) and decay (Large $T_{50,\ deg}$) rates for large cells. Red dashed line indicates the y = x line. Blue dots indicate binned averages, and error bars mark the 95% confidence interval. N = 2656 proteins. **B)** and **C)** Comparison of protein turnover obtained from our study (both small and large cells) against those from a previously published study in HeLa cells [S3]. N = 1882 proteins for both plots. **D)** and **E)** Biological replicate comparison of protein turnover derived from small and large cells. N = 2018 proteins for both plots. **F**) Distribution of proteins both present and absent in the protein turnover dataset as compared against the size-scaling proteome dataset (**Figure 1**). Lowly expressed proteins tend to be excluded from the turnover dataset. **G)H)** Protein turnover is not correlated with expression level in small and large cells. **I**) Change in protein turnover is not correlated with expression level. For **F)G)H) and I)**, MS1 intensity was used as an approximation of absolute mass fraction and thus, the expression level. For all panels except **F)**, blue dots are averages of equal sized bins and error bars represent the 95% confidence intervals.

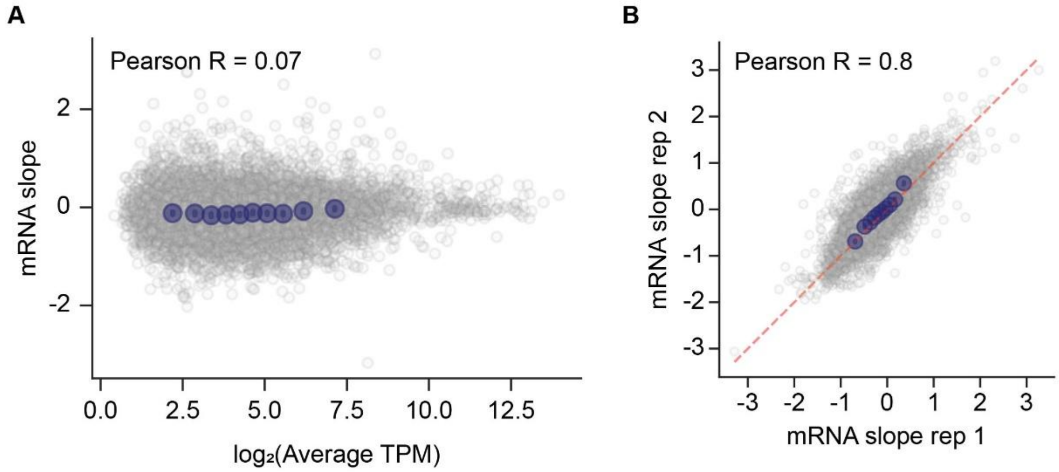
A
Pearson R = 0.07
mRNA slope
log₂(Average TPM)
B
Pearson R = 0.8
mRNA slope rep 2
mRNA slope rep 1

Figure S4. Size-dependent transcriptomic scaling does not depend on expression level, related to Figure 4.

A) Size-dependent changes in mRNA concentration do not correlate with absolute expression levels. mRNA slope values were compared against the genes' respective basal TPM values in medium-sized cells. N = 10139 genes. **B**) Comparison of mRNA slopes from two replicate experiments. Each replicate slope is itself an average of the slopes calculated from samples following DMSO or doxycycline induction. N = 10139. For both **A)** and **B)**, blue dots are averages of equal sized bins

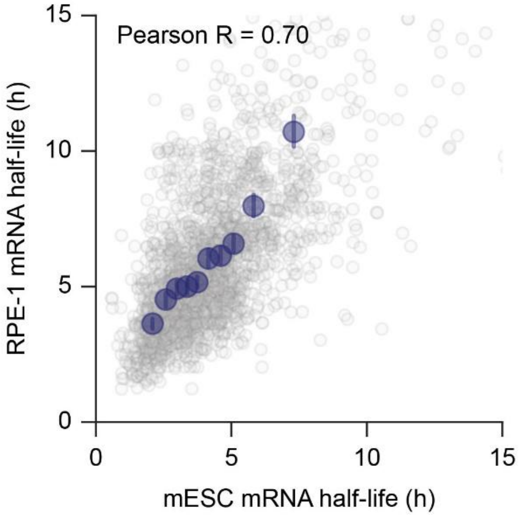
Pearson R = 0.70
RPE-1 mRNA half-life (h)
mESC mRNA half-life (h)
15
10
5
0
0
5
10
15

Figure S5. mRNA half-lives in RPE-1s correlate with those in mESCs, related to Figure 5. Comparison of mRNA half-life measured by this study and those from mESCs from a previously published study using SLAMseq [S4]. Average mRNA half-life of middle-sized cells were used for RPE-1 mRNA half-life. Mouse genes were humanized using the orthologs database from Mouse Genome Informatics. Blue dots are the binned averages of equal number of points, and error bars represent the 95% confidence intervals. N = 2145 genes.

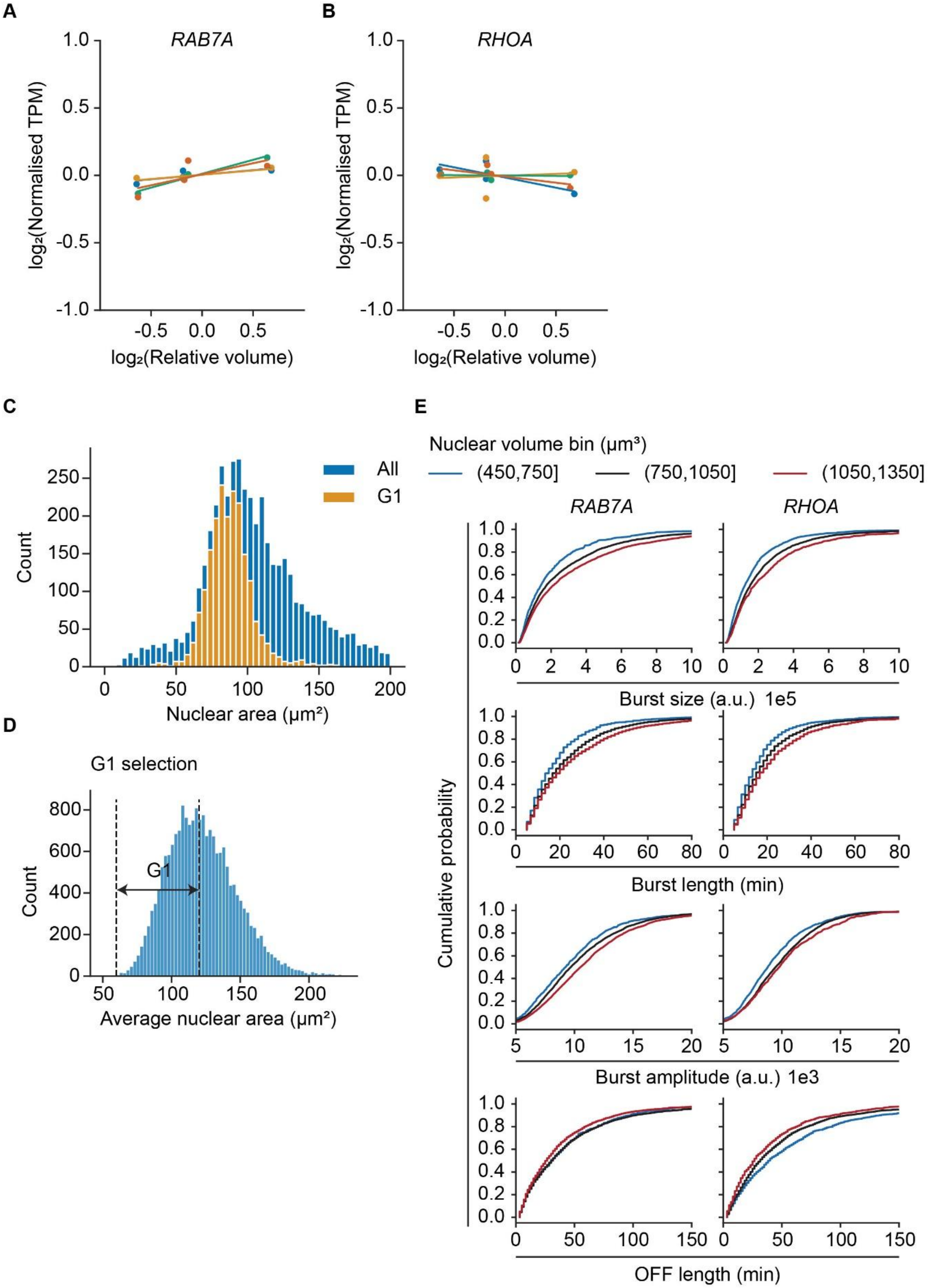
A
B
RAB7A
RHOA
log₂(Normalised TPM)
log₂(Relative volume)
C
All
G1
Count
Nuclear area (μm²)
D
G1 selection
G1
Average nuclear area (μm²)
E
Nuclear volume bin (μm³)
(450,750]
(750,1050]
(1050,1350]
Cumulative probability
Burst size (a.u.) 1e5
Burst length (min)
Burst amplitude (a.u.) 1e3
OFF length (min)

Figure S6. Transcriptional burst size and ON time scale with cell size, related to Figure 6. Size scaling mRNA concentrations for **A)** *RAB7A* and **B)** *RHOA* as measured by RNAseq. Individual points mark the transcript's gene-specific mean-normalized TPM measured for a given size, and separate colors denote different replicates (N = 4 replicates). **C)** Nuclear area distribution of HBEC-kt3 cells with endogenous *RAB7A* tagging of MS2 stem loops. G1 population distribution is highlighted to indicate G1 cells' area distribution. Cells stained for DNA content with Hoechst were imaged with a widefield epifluorescence microscope separately from live-imaging experiments to determine nuclear size distribution of G1 cells. Nuclear area was used as a proxy for cell size. **D)** Window of selection of HBEC-kt3 cells tagged with endogenous *RAB7A* tagging of MS2 stem loops for G1. These cells were selected for further downstream analysis of burst dynamics for this study. **E)** Cumulative distribution function plot of different burst parameters for genes *RAB7A* and *RHOA* binned by increasing cell size. Plots have been enlarged to highlight size-dependent changes in burst parameters.

Supplemental references

1. Lanz, M.C., Zatulovskiy, E., Swaffer, M.P., Zhang, L., Ilerten, I., Zhang, S., You, D.S., Marinov, G., McAlpine, P., Elias, J.E., et al. (2022). Increasing cell size remodels the proteome and promotes senescence. Mol. Cell *82*, 3255-3269.e8. https://doi.org/10.1016/j.molcel.2022.07.017.
2. Lanz, M.C., Zhang, S., Swaffer, M.P., Ziv, I., Götz, L.H., Kim, J., McCarthy, F., Jarosz, D.F., Elias, J.E., and Skotheim, J.M. (2024). Genome dilution by cell growth drives starvation-like proteome remodeling in mammalian and yeast cells. Nat. Struct. Mol. Biol. *31*, 1859–1871. https://doi.org/10.1038/s41594-024-01353-z.
3. Zecha, J., Meng, C., Zolg, D.P., Samaras, P., Wilhelm, M., and Kuster, B. (2018). Peptide Level Turnover Measurements Enable the Study of Proteoform Dynamics. Mol. Cell. Proteomics *17*, 974–992. https://doi.org/10.1074/mcp.RA118.000583.
4. Herzog, V.A., Reichholf, B., Neumann, T., Rescheneder, P., Bhat, P., Burkard, T.R., Wlotzka, W., von Haeseler, A., Zuber, J., and Ameres, S.L. (2017). Thiol-linked alkylation of RNA to assess expression dynamics. Nat. Methods *14*, 1198–1204. https://doi.org/10.1038/nmeth.4435.
